# Effectiveness of a School-Based Culinary Programme on 9- and 10-Year-Old Children’s Food Literacy and Vegetable, Fruit, and Breakfast Consumption

**DOI:** 10.3390/nu15061520

**Published:** 2023-03-21

**Authors:** Charlotte Labbé, Stephanie Ward Chiasson, Jérémie B. Dupuis, Claire Johnson

**Affiliations:** 1 École des Sciences des Aliments, de Nutrition et d’Études Familiales, Université de Moncton, Moncton, NB E1A 3E9, Canada; ecl7042@umoncton.ca; 2 Vice-Rectorat à l’Enseignement et à la Recherche, Université de Moncton, Moncton, NB E1A 3E9, Canada; jeremie.dupuis@umoncton.ca; 3 École des Hautes Études Publiques, Université de Moncton, Moncton, NB E1A 3E9, Canada; claire.johnson@umoncton.ca

**Keywords:** children, cooking, feeding behaviour, health literacy, nutritional sciences/education

## Abstract

School-based culinary courses may increase children’s food literacy and improve their eating behaviours. This study assessed the impact of a school-based culinary programme on 9- and 10-year-old students’ food literacy and vegetable, fruit, and breakfast consumption. This cluster quasi-experimental trial compared 88 grade 4 and 5 students who participated in the *Apprenti en Action* programme to 82 students who did not. Students’ food literacy and eating behaviours were assessed with a self-administered questionnaire. The programme’s impact on vegetable and fruit consumption, cooking skills, food skills, and food knowledge was measured using MANOVA, and the odds of eating breakfast at least five times per week were assessed with logistic regression. Students who participated in the programme reported a greater increase in their cooking skills (*p* = 0.013) and food knowledge (*p* = 0.028) than students in the control group. No effect was found on food skills and vegetables, fruit, and breakfast consumption (*p*-values > 0.05). Boys improved their cooking skills (*p* = 0.025) and food knowledge (*p* = 0.022), but girls did not. The programme improved students’ cooking skills and food knowledge, especially among boys; however, modifications are needed to improve students’ food skills and eating behaviours.

## 1. Introduction

Adopting a healthy diet and eating behaviours in childhood is essential for optimal growth and cognitive development [1]. It may also reduce the risk of developing chronic diseases (e.g., cardiovascular disease, cancer, type 2 diabetes) later in life [2,3,4]. However, in many developed countries, school-aged children’s diets are low in vegetables and fruits and unhealthy eating behaviours, such as skipping breakfast, are common [5,6,7]. In Canada, only 45% of children between the ages of 9 and 13 years reported consuming at least five servings of vegetables and fruits recommended by the World Health Organization [8,9]. In the Canadian province of New Brunswick, a 2016 provincial survey found that 51% of boys and 54% of girls in kindergarten to grade 5 reported eating five or more vegetables and fruits the previous day [10]. The same survey also found that only 69% of boys and 71% of girls reported eating breakfast daily [10].

Increasing children’s vegetable and fruit intake and adopting healthy eating behaviours is essential; however, doing so is complex. As Bandura’s social cognitive theory describes, human behaviours are influenced by numerous cognitive, environmental, and behavioural factors [11]. Although increasing children’s awareness and knowledge of how to eat healthily is critical, recent studies have suggested that it is not enough to lead to significant changes in eating behaviours [12]. Therefore, researchers have proposed that improving children’s food literacy may be a more promising approach [5,12,13,14].

The definition of food literacy, a relatively new and emerging concept, has evolved over the past two decades [15,16,17]. Although numerous definitions of food literacy exist, Vidgen and Gallegos’ (2014) is the most cited [18]. The authors define it as “[…] a collection of inter-related knowledge, skills and behaviours required to plan, manage, select, prepare and eat food to meet needs and determine intake” [19] (p. 54). Based on this definition, food literacy can be interpreted as having three major components: adequate food skills (i.e., being able to plan, manage, and select food), cooking skills (i.e., being able to prepare food), and the knowledge required to make healthy food choices and adopt healthy eating behaviours (e.g., eating vegetables and fruits, having breakfast daily) [20]. Recent studies have found that greater food literacy has been associated with healthier eating behaviours among children and adolescents [5,12,13,21,22]. For example, a cross-sectional study among 1054 adolescents in New Brunswick, Canada, found that better cooking and food skills are associated with greater vegetable and fruit consumption and healthier eating behaviours among girls and boys [21]. Another study among 803 Iranian elementary students reported that greater food and nutrition literacy scores are associated with healthier eating behaviours [23].

Since previous studies have found a positive relationship between food literacy and healthy eating behaviours, various interventions have been developed to increase children’s and adolescents’ cooking skills, food skills, and nutrition knowledge [5,13,20]. While the impact of these interventions on food literacy, diet, and eating behaviours is variable, those that were most effective had multiple components, such as hands-on culinary programmes combined with nutrition education and taste-testing sessions [24,25]. Since children spend most of their waking hours at school, engaging them in multi-component, school-based culinary courses may help increase their food literacy and improve their eating behaviours. Specifically, these courses may help them learn how to cook and prepare foods (cooking skills), plan meals, follow a recipe, or read nutrition labels (food skills), all the while learning the basics of nutrition and how to make healthier food choices (food knowledge). However, few studies have assessed the effect of such programmes on the multiple components of food literacy, with food skills being the least studied. Therefore, the true impact of school-based culinary courses on students’ food literacy remains unclear.

While previous school-based culinary programmes have shown promising results, some studies have suggested that their impact may be influenced by gender [20,26,27,28,29]. These observations may be due to differences in nutritional knowledge, dietary intake, food preferences, and eating behaviours between girls and boys [5,13,30,31,32,33,34,35,36]. For instance, girls have been found to have greater intakes of vegetables and fruit [33] and have better nutritional knowledge than boys [31]. Further, a quasi-experimental study among adolescents found that while girls improved their cooking and food skills after taking an elective semester-long high school cooking course, boys only improved their cooking skills [20]. Such findings suggest that assessing the impact of school-based culinary interventions through a gender lens is warranted. However, few studies have done so thus far.

This study assessed whether a school-based culinary programme could improve boys’ and girls’ food literacy, including cooking skills, food skills, and knowledge; increase their consumption of vegetables and fruits; and increase how often they eat breakfast. Findings from this study help strengthen the existing body of evidence related to the role that school-based culinary programmes play in improving students’ food literacy and eating behaviours and offers insight into whether girls and boys respond equally to these types of interventions. This study also provides valuable information to schools that are considering offering culinary programmes to their students and may help justify integrating such programmes into the schools’ curriculum.

## 2. Materials and Methods

### 2.1. Study Design

A cluster quasi-experimental study was conducted to assess the impact of a school-based culinary programme called *Apprenti en Action* on students’ food literacy and eating behaviours (trial registration #NCT05278377). Students in three schools following the programme (experimental group) were compared to those enrolled in three control schools. The 6-week intervention was preceded and followed by data collection; baseline data were collected in April/May 2022, and endpoint data were collected in June 2022. Due to the nature of the study, blinding was not possible.

### 2.2. Participants and Recruitment

**Figure 1:**
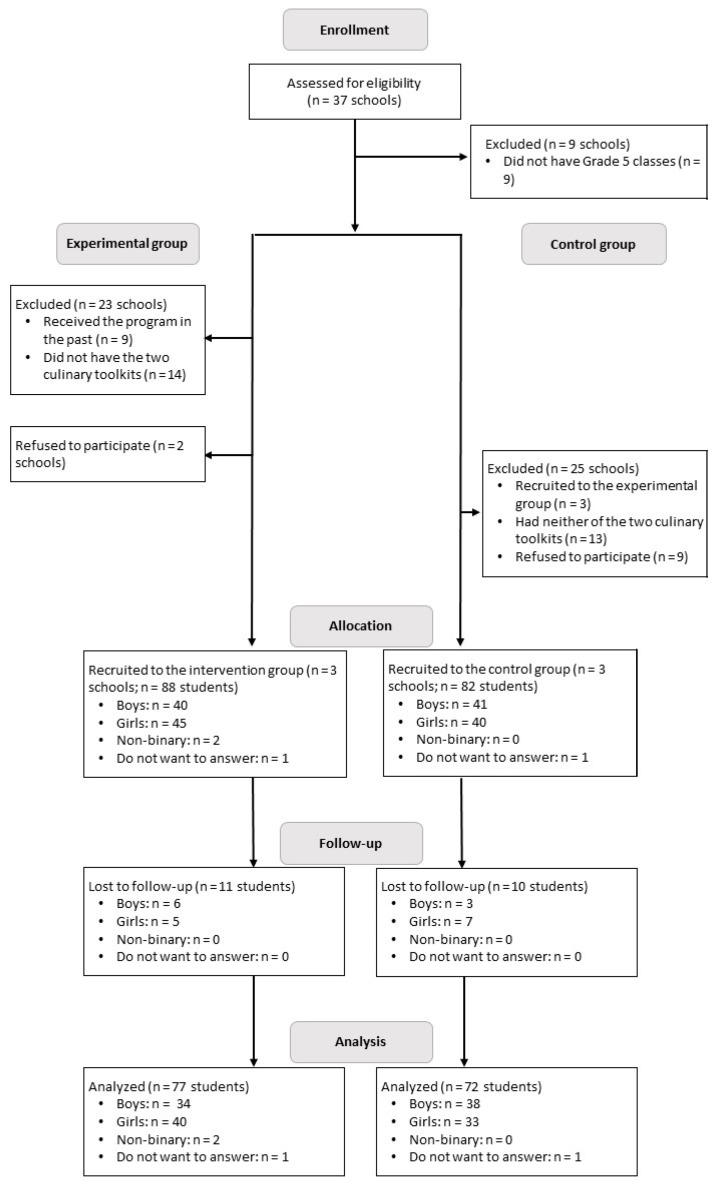
Flow diagram of participants at each stage of the study.

Six public francophone elementary schools in the District scolaire francophone Sud (DSFS) in New Brunswick, Canada, were recruited for this study. To achieve the desired sample size for this study, slightly different inclusion criteria were needed to select experimental and control schools. To participate in the experimental group, schools had to have grade 4 and 5 classes, have purchased the two culinary toolkits that are required for the programme (one that contains food preparation equipment and the other that includes cooking equipment), and not have previously participated in the programme. Of the 37 elementary schools in the DSFS, 9 did not offer grade 5 classes, and among those that did, 14 did not have both toolkits (Figure 1). Only 5 of the remaining 14 schools were eligible to participate in the experimental group since 9 had previously followed the programme. Although it was initially planned to recruit all five schools, two declined to participate due to staffing issues. Therefore, three schools were recruited to follow the *Apprenti en Action* programme. All grade 5 students and those in mixed grade 4–5 classes were eligible and invited to participate in the study.

For the control group, schools had to have grade 4 and 5 classes and have purchased at least one of the two culinary toolkits. While culinary toolkits were not essential for this group, it was considered a sign that schools were interested in the programme and would be ready to receive it once the study was completed. This criterion was also used to ensure similarity between schools in each group. Of the 12 schools that were eligible for the control group, 3 were randomly selected and invited to participate. If a school declined, another school was chosen randomly. In the end, three schools agreed to participate, three did not respond to the invitation, and six refused due to lack of time, staffing issues, or lack of interest in offering the programme at that time. All grade 4 and 5 students in the three recruited schools were eligible and invited to participate. Signed informed consent and assent were obtained from parents and students, respectively. This study received ethics approval from the Comité d’éthique de la recherche avec les êtres humains at the Université de Moncton (#2122-006).

### 2.3. Intervention

*Apprenti en Action* is a school-based culinary programme developed and delivered by the Healthy Eating and Social Entrepreneurship Coordinator at the DSFS. The programme consists of 45 min culinary workshops offered once a week, at school, during school hours, for 6 weeks. During these workshops, students are taught the basics of healthy eating, food, and nutrition; how to read and follow recipes; how to prepare and cook various foods using different culinary techniques; and how to apply food safety practices. Culinary demonstrations and culinary art were also embedded within the programme, and students were invited to eat what they and their peers had prepared.

The programme requires schools to purchase two culinary toolkits valued at CAD 2500 for both, which contain the necessary equipment to prepare and cook foods (e.g., cutting boards, paring knives, induction plates, pans, pots, peelers, melon ballers, and measuring cups). Funding was available through community programmes to any school needing financial assistance to purchase these toolkits. *Apprenti en Action* is designed for students from kindergarten through grade 8, with age-appropriate themes and activities based on children’s developmental abilities. For grade 5 students, the programme focuses primarily on preparing healthy breakfasts. Students learn how to make applesauce, granola, smoothie bowls, and French toast as well as how to cook eggs. The programme also includes a curriculum of activities for other grade levels. However, the breakfast theme is only offered to grade 5 students or those in mixed grade 4–5 classes and was the only curriculum provided in schools at the time of this study.

### 2.4. Data Collection

Students completed a self-administered online questionnaire comprising 35 questions to assess vegetable, fruit, and breakfast consumption; cooking skills; food skills; and nutrition knowledge. The questionnaire was administered in late April/early May 2022 (baseline) and June 2022 (endpoint). The questionnaire took students approximately 20–25 min to answer.

The questionnaire was inspired by a variety of existing tools, including validated questionnaires from Skeaff et al. (2020), Dean et al. (2021), Lavelle et al. (2017), and the New Brunswick Student Wellness Survey [37,38,39,40]. Additional questions were also developed in collaboration with the Healthy Eating and Social Entrepreneurship Coordinator of the DSFS to ensure that all variables of interest were measured. Once the questionnaire was developed in English, back-translation was conducted to translate it into French [41]. The final draft of the questionnaire was piloted in March 2022 with two francophone students aged 9 and 10 years.

#### 2.4.1. Cooking Skills

Cooking skills include all skills needed to prepare and cook foods, including cooking methods and food preparation techniques [38]. In this study, students were asked to report their confidence level in using various cooking methods, food preparation techniques, and kitchen tools; their ability to apply food safety practices while cooking; and their ability to prepare breakfast. Students’ cooking skills were measured using 19 questions, which were adapted from Skeaff et al.’s (2020), Dean et al.’s (2021), and Lavelle et al.’s (2017) questionnaires [37,38,40]. In some instances, questions were adapted to the Canadian context (e.g., dietary guidelines, foods available in Canada) or the specific skills learned through the *Apprenti en Action* programme. Additional questions were also developed in collaboration with the Healthy Eating and Social Entrepreneurship Coordinator to measure other essential skills emphasized throughout the programme (e.g., how to hold food when cutting it, where to place it on a cutting board).

Among the 19 questions, 2 used a Likert scale. The first one, adapted from Skeaff et al.’s (2020) questionnaire, asked students to rate their ability to make hard-boiled eggs, French toast, smoothies or smoothie bowls, and applesauce. Response options for this question were “I have never done it” (0 points), “I can’t do it at all” (0 points), “I can do it with a lot of help” (1 point), “I can do it with a little help” (2 points), and “I can do it all on my own” (3 points) [40]. The second question, adapted from Lavelle et al.’s (2017) questionnaire, asked students to report how good they were at performing seven different cooking skills (i.e., cutting food, peeling fruit, breaking an egg, mixing and stirring food, mixing food to make them smooth, boiling or simmering food, and baking in the oven). Response options ranged from 1 (very poor) to 7 (very good). A score of 0 was awarded if the student answered “never/rarely” [38].

The other 17 questions were multiple-choice or multiple-answer questions. They were developed in collaboration with the Healthy Eating and Social Entrepreneurship Coordinator or inspired/adapted from questionnaires by Skeaff et al. (2020) and Dean et al. (2021) [37,40]. These questions asked students about cooking hard- and soft-boiled eggs as well as applesauce (3 questions, 4 points) [40] and which kitchen equipment they should use to measure dry and liquid ingredients (1 question, 6 points). They were also asked to match kitchen equipment to food preparation techniques (e.g., cut/slice, mix/stir, peel, measure; 2 questions, 9 points) [40] and to identify, with the help of images, proper food preparation techniques (11 questions, 11 points) [37]. For all questions, missing answers were awarded a score of 0. After adding the score of all the questions related to cooking skills, this variable had a score ranging from 0 to 91 points.

#### 2.4.2. Food Knowledge

Food knowledge represents facts and information about food, nutrition, and health [42]. In this study, students were asked questions that tested their knowledge of food safety, safe food-handling practices, and how to prepare a balanced meal. Questions related to knowledge were inspired by Skeaff et al.’s (2020) questionnaire [40]. Since the original questionnaire was developed for students in New Zealand, questions were culturally adapted and modified to measure what was taught during the programme. A total of six questions assessed students’ knowledge. Four were multiple-choice questions, where a score of 1 was assigned for a correct answer (0 for a wrong answer) [40]. The other two were multiple-answer questions; the first had up to three correct answers, and the other had up to four [40]. Each correct answer was awarded 1 point. For any of the six questions, a score of 0 was given if an answer was missing. In total, this variable’s score ranged from 0 to 11 points.

#### 2.4.3. Food Skills

Food skills are the knowledge and skills required to choose and prepare food using available resources and to cook balanced meals [38,43,44]. In this study, food skills included knowing how to prepare certain foods without a recipe, plan meals, follow a recipe, prepare a balanced breakfast, and help with groceries. Students’ food skills were assessed using questions from Skeaff et al.’s (2020) and Lavelle et al.’s (2017) questionnaires, which were culturally adapted or modified to represent specific programme objectives [38,40]. Two were multiple-choice questions [40]; 1 point was awarded for the correct answer, and 0 points were awarded for the wrong answer or if it was missing. A third question used a 5-point Likert scale [38]. This question asked students to rate how well they performed seven different food skills on a scale of 1 to 7, where 1 was “very bad” and 7 was “very good” [38]. A score of 0 was awarded if a student answered “never/rarely” or if an answer was missing. When adding the score of all three questions, a score ranging from 0 to 53 points was possible for this variable.

#### 2.4.4. Vegetable and Fruit Consumption

Students’ consumption of vegetables and fruits was measured using two questions from the New Brunswick Student Wellness Survey [39]. These questions asked, “Yesterday, how often did you eat fruit, fresh, cooked, frozen, canned or dried? (Don’t count: fruit juice, fruit rolls or fruit candy)” and “Yesterday, how often did you eat vegetables, fresh, cooked, frozen or canned?” A score of 0 (none or “I don’t know”) to 7 (7 or more times) was awarded to each of these questions for a total score ranging from 0 to 14 points [39].

#### 2.4.5. Frequency of Breakfast Consumption

The frequency of breakfast consumption was measured using one question from the New Brunswick Student Wellness Survey, which asked, “Last week, how many times did you eat breakfast”? [39]. A score ranging from 0 (none) to 7 (7 days of the week) was possible for this question [39]. Data were considered missing if a student answered, “I do not know”.

#### 2.4.6. Confounding Variables

In this study, students’ age, gender, ethnicity, and their family’s socioeconomic status were considered potential confounding variables as they have been shown to influence people’s diets [33,45,46], as well as cooking and food skills [47]. Sociodemographic data, including parental education level, household income, and the child’s ethnicity, were collected via a short parental questionnaire that followed the consent form. Children’s age, gender, and school were obtained through the student self-administered food literacy questionnaire.

To determine the child’s socioeconomic status, the mean education level (1 = high school diploma, GED, or less; 2 = post-secondary certificate or community college diploma; 3 = university certificate or diploma below bachelor’s degree; 4 = bachelor’s degree; 5 = university certificate or diploma above a bachelor’s degree. such as a master’s degree, professional degree, or doctorate; 6 = other) of both parents was calculated and added to the household income (1 = less than CAD 5000, up to 14 = CAD 150,000 or more) [48,49]. Children’s ethnicity was categorized as “White”, “Black”, or “Other”, where the latter included children who identified as South Asian, Chinese, Filipino, Latin American, Arab, Southeast Asian, West Asian, Korean, Japanese, or another ethnic origin [50]. Students’ age at the beginning of the study was calculated in months based on their reported birth date. The students’ school was included as a confounding variable to control for the intraclass correlation of students within the same school. Finally, since there are differences in food intake, food knowledge, and food skills between boys and girls [33,34,36], students also reported whether they identified as a “boy”, a “girl”, or “non-binary”.

### 2.5. Statistical Analysis

Data analyses were conducted in SPSS (version 28.0.1.1). Only students who completed both pre- and post-questionnaires were included in the analyses. Each variable’s distribution was examined to identify potential outliers. While no data transformations were required for vegetable and fruit consumption, or food knowledge, a square root transformation was performed for cooking skills and food skills to normalize the distribution. Since conventional transformation methods failed to normalize the breakfast consumption data, this variable was dichotomized. As such, students who reported consuming breakfast five times or more in the previous week were considered regular breakfast eaters, while those who reported eating breakfast less than five times in the past week were regarded as irregular breakfast eaters. Baseline differences for all variables were examined between groups using *t*-tests or chi-square tests.

Multivariate analysis of variance (MANOVA) was performed to assess the impact of the *Apprenti en Action* programme on vegetable and fruit consumption, cooking skills, food knowledge, and food skills. Logistic regression was used to measure the programme’s impact on the odds of consuming breakfast regularly. For the MANOVA and logistic regression, a group-by-time interaction effect was used as the primary independent variable to control for differences in the outcome variables at baseline between both groups. Confounding variables, including students’ age, gender, ethnicity, school, and family socioeconomic status, were controlled for in the MANOVA and logistic regression. Since only 1.2% of students identified as non-binary, separate gender analyses were performed for boys and girls only. Statistical significance was considered at *p* < 0.05.

## 3. Results

A total of 170 students (88 in the experimental group and 82 in the control group) completed the questionnaire in late April/early May 2022 (Figure 1). Of those students, 149 (77 in the experimental group and 72 in the control group) answered the same questionnaire after the programme (June 2022). Some participants declined to participate or were absent between the pre- and post-assessments, which explains why participation rates dropped by 12%. Despite the loss at follow-up, the final sample size was slightly greater than the one initially calculated using G*Power, which recommended a sample of 128 students for an alpha error probability of 0.05, an effect size of 0.25, and a power of 80%.

The baseline characteristics of participants in the experimental and control groups are shown in Table 1. On average, children were 10.6 years old, consisted of 50% girls, and 75.3% were Caucasian. Sociodemographic characteristics were similar between students in the experimental group and those in the control group, except that children in the experimental group were slightly older (*p* < 0.01) and had greater cooking skills (*p* < 0.05). At baseline, girls’ food knowledge and food skills were statistically greater than boys’ in both experimental and control groups (*p* < 0.05). While there was no difference between girls’ and boys’ cooking skills in the control group, girls had greater cooking skills than boys in the experimental group (*p* < 0.05).

Overall, MANOVA showed that students in the experimental group had, on average, an 11.0 greater point increase in cooking skills and a 0.94 greater point increase in food knowledge compared to students in the control group (Table 2). No significant differences in changes in food skills and vegetable and fruit consumption were found between the experimental and control groups. Logistic regressions showed that students in the experimental group did not increase their odds of eating breakfast regularly (five times per week or more) compared to students in the control group (Table 3). Gender was a significant confounding variable in all models (all *p*-values < 0.05), except for the odds of eating breakfast regularly. Students’ age was a significant confounding variable for cooking skills (*p* = 0.026), ethnicity was significant for food knowledge (*p* = 0.035), and socioeconomic status was significant for the odds of eating breakfast regularly (*p* = 0.011).

Gender analyses showed that boys in the experimental group had, on average, a 12.04 greater point increase in cooking skills and a 1.45 greater point increase in their food knowledge than boys in the control group (Table 2). No significant differences in changes in boys’ food skills, vegetable and fruit consumption, or their odds of eating breakfast regularly were found between the two groups. Finally, the programme was not found to have significantly impacted girls’ cooking skills, food skills, food knowledge, vegetable and fruit consumption, or their odds of eating breakfast regularly. Among boys, students’ age was a significant confounding variable for cooking skills (*p* = 0.015). In contrast, ethnicity and socioeconomic status were significant confounding variables for boys’ odds of eating breakfast regularly (*p* = 0.004 and *p* = 0.016, respectively). Ethnicity was the only significant confounding variable for girls’ food knowledge (*p* = 0.016).

## 4. Discussion

This study assessed the impact of a school-based culinary programme on boys’ and girls’ food literacy, including cooking skills, food knowledge and skills, their consumption of vegetables and fruits, and how often they eat breakfast. It is one of the few to simultaneously assess the effectiveness of a school-based culinary programme on three components of students’ food literacy, including food skills, and to examine its impact on girls and boys separately. This study found that the *Apprenti en Action* programme improved students’ cooking skills and food knowledge but not their food skills. Further, gender analyses suggest that these changes were primarily due to improvements among boys. Although the *Apprenti en Action* programme was unsuccessful at improving students’ vegetable and fruit consumption or increasing their odds of eating breakfast regularly, previous studies have shown that greater cooking skills are associated with healthier eating behaviours, including vegetable and fruit consumption [21].

Improvements in students’ cooking skills following their participation in the *Apprenti en Action* programme were expected, as the programme focused primarily on teaching them how to cook. These findings are consistent with other Canadian studies’ findings [20,27,51]. For example, Zahr and Sibeko (2017) found that grade 4 and 5 students’ self-reported ability to measure ingredients, cut vegetables and fruit, and prepare a balanced meal on their own improved after participating in a programme that included cooking classes and taste-testing activities [51]. Improving children’s cooking skills is important. They have been linked to a greater interest in cooking meals [52] and healthier eating behaviours, such as increased vegetable and fruit consumption and decreased consumption of convenience foods [21,53,54]. In 2015, 57.2% of the energy intake of Canadians aged between 9 to 13 years came from ultra-processed foods (e.g., pre-prepared pizzas and frozen dishes, candies, desserts) [55]. Knowing how to cook may enable school-aged children to prepare (e.g., peel, pare, slice) or cook various vegetables and fruits to make them more palatable and enjoyable, while reducing their reliance on convenience foods. Children who can participate in meal preparation also tend to have healthier diets [56,57] and are more likely to eat what they have prepared [58].

Similarly to previous studies, the *Apprenti en Action* programme increased students’ food knowledge [22,27,59,60,61,62,63,64]. Although this outcome was mostly found in programmes that included theory-based learning, nutrition education was integrated into the culinary workshops of the *Apprenti en Action* programme. For example, when soy butter was used in one of the recipes, information about this product was provided to students while they used it. This finding suggests that nutrition education taught during activity-based learning can improve students’ food knowledge and that theory-based learning may not be essential for all food literacy programmes. Findings from Liquori et al.’s (1998) study support this hypothesis. The authors observed that grade 4 to 6 students saw greater increases in food knowledge when they participated in cooking workshops combined with nutrition education than when they only received theory-based nutrition education [65]. This finding is important since better food knowledge is associated with healthier eating behaviours (e.g., increased vegetable and fruit consumption and greater diversity in food intake) [66,67,68]. However, food knowledge alone may not be enough to significantly change children’s eating behaviours [12]. Therefore, increasing food knowledge should be seen as a first step toward improving food literacy rather than being the only desired outcome.

Similar to cooking skills, better food skills have also been associated with healthier diets or eating behaviours, including greater vegetable, fruit, and breakfast consumption among children, adolescents, and adults [21,69,70]. However, this study found no impact of the *Apprenti en Action* programme on students’ food skills. Other school-based culinary programmes have also struggled to improve this food literacy component [51,71]. Many food skills are complex and require that children have adequate nutrition knowledge and cooking skills. For example, preparing a nutritiously balanced meal requires that children know how to select healthy foods, how to prepare and cook them, and how to combine them based on their nutritional composition. Therefore, children may need to acquire a certain level of cooking knowledge and competency before significant improvement in food skills can be observed. Compared to cooking skills, some food skills may also be more difficult to acquire during a school-based programme since students need to practice them at home. For example, it may be difficult for children to learn how to plan a meal or use a grocery list while shopping if these practices are not common in their households. Therefore, it may be helpful for food-literacy-promoting programmes to provide parents with information about the benefits of using food management strategies (e.g., menu planning with their children) and how to involve their children in these activities.

Contrary to expectations, the *Apprenti en Action* programme did not increase students’ vegetable and fruit consumption or their odds of eating breakfast at least five times per week. Previous interventions have found mixed results, with some studies reporting positive effects on vegetable and fruit [72,73] or breakfast [61] consumption, others finding none [74,75,76], and some noting positive effects on one type of food (fruits or vegetables) but not the other [26,77]. These conflicting findings may be due to the fact that children’s eating behaviours are influenced by internal and external factors, such as personal preferences, cultural beliefs and values, lack of time to eat in the morning, exposure to intensive food marketing strategies, and the types of foods available and accessible in the home and school food environment [33,78,79]. According to Birnbaum et al. (2002), combining classroom activities such as culinary classes with environmental changes (e.g., increasing vegetable and fruit availability and accessibility at school, encouraging parents to make healthy food choices at home) may be a more promising approach to help promote vegetable and fruit consumption [73]. Thus, adding such interventions to the *Apprenti en Action* programme may help improve its impact on students’ eating behaviours. Additions to the curriculum may also be warranted, such as emphasizing building students’ food skills (e.g., meal planning). so that they can overcome common barriers to eating vegetables and fruits and consuming breakfast.

Interestingly, this study found that the *Apprenti en Action* programme was effective at increasing boys’ cooking skills and food knowledge but had no effect on girls. Most studies have observed the opposite, noting significantly greater improvements in girls’ food literacy or eating behaviours than in boys’ [20,26,29]. This study’s findings may be explained by the fact that girls’ cooking skills and food knowledge at baseline were significantly greater than boys’. Previous studies have also shown that girls tend to have greater nutrition knowledge and better cooking skills than boys [31,32,80], making them more likely to participate in cooking courses or programmes [20]. Since girls had better cooking skills and food knowledge at baseline than boys, there may have been a greater opportunity for improvement among boys than girls. Our finding that the *Apprenti en Action* programme significantly impacted boys’ cooking skills and food knowledge reiterates the importance of providing such programmes to all students, not just those who show interest.

To increase the programme’s impact, especially on eating behaviours (i.e., vegetable and fruit consumption, eating breakfast) and food skills, it may need to be offered over a longer period and include additional components, such as parent newsletters or activities to be completed at home [61,62,77,81]. School-based environmental changes or interventions may also need to be implemented simultaneously, such as making vegetables and fruits more readily accessible (e.g., providing free vegetables and fruits at snack time), offering universal breakfast programmes, and engaging students in school gardens [26,62,73,77]. In addition, the programme may need to provide various culinary activities to accommodate different skill levels among students. For example, recipes with more advanced culinary techniques could be offered to students with greater cooking skills. Culinary programmes, such as *Apprenti en Action,* should also ensure that food skills are adequately taught, as they have been shown to have a greater impact on diet quality than cooking skills [69,82]. Finally, it is recommended that culinary programmes be integrated into the schools’ curriculum. This would ensure their viability and allow all students to participate.

The strengths of this study include assessing multiple components of food literacy (cooking skills, food knowledge, and food skills) in a real-world context, including confounding variables (students’ age, gender, ethnicity, and school, as well as their family’s socioeconomic status) in the analysis and evaluating the programme’s impact on boys and girls separately. However, some limitations need to be acknowledged. Although the questionnaire used in this study was developed based on previously validated questionnaires, the psychometric properties of the final tool were not tested. Since most questions from the previous questionnaires were modified and adapted, it is likely that their psychometric properties also changed. Although the questionnaire was not validated, the tool reflects programme objectives and what was taught in the *Apprenti en Action* programme. In addition, the questionnaire was self-administered, which could have led to social desirability bias. Some students may have overestimated their food and cooking skills and vegetable, fruit, or breakfast consumption. The short duration of the programme is also a limitation of this study. It may not have been long enough to influence more complex eating behaviours, such as vegetable, fruit, and breakfast consumption.

## 5. Conclusions

This study is one of the first to assess the impact of a culinary programme on three major components of food literacy among boys and girls separately. Our findings suggest that the cooking skills and food knowledge of grade 4 and 5 students can be significantly improved after participating in a 6-week culinary programme and that boys may benefit the most. Although the programme was ineffective at increasing vegetable and fruit consumption or the odds of eating breakfast regularly, culinary programmes, such as *Apprenti en Action*, may positively impact those outcomes in the long term by helping children increase their food literacy. Longitudinal studies are required to confirm this hypothesis. Future studies should also consider how culinary programmes can be adapted to ensure that both boys and girls benefit from such interventions and how they can integrate activities that would help increase food skills.

## Figures and Tables

**Table 1:** Descriptive characteristics of the study sample at baseline.

	Control (n = 82)	Experimental (n = 88)	*p*-Value
	n (%)	n (%)	
Age (years), mean ± SD	10.40 ± 0.58	10.85 ± 0.43	**<0.01**
9 years old	23 (28.0%)	1 (1.1%)	
10 years old	46 (56.1%)	59 (67.0%)	
11 years old	13 (15.9%)	25 (28.4%)	
12 years old	0 (0%)	3 (3.4%)	
Gender			0.552
Boys	41 (50.0%)	40 (45.5%)	
Girls	40 (48.8%)	45 (51.1%)	
Other	1 (1.2%)	3 (3.4%)	
Ethnicity			0.085
Caucasian	60 (73.2%)	68 (77.3%)	
Black	1 (1.2%)	5 (5.7%)	
Other	12 (14.6%)	6 (6.8%)	
Socioeconomic status			0.116
Low	3 (3.7%)	4 (4.5%)	
Medium	47 (57.3%)	36 (40.9%)	
High	24 (29.3%)	37 (42.0%)	
	**Mean (SD)**	**Mean (SD)**	
Cooking skills (0 to 91 pts)	27.95 (12.77)	36.52 (20.10)	**0.009**
Food knowledge (0 to 11 pts)	5.41 (1.61)	5.34 (1.98)	0.286
Food skills (0 to 53 pts)	12.79 (9.32)	15.57 (12.57)	0.791
Vegetable and fruit consumption (0 to 14 pts)	6.15 (4.37)	5.50 (4.12)	0.325
	**%**	**%**	
Percentage of students eating breakfast regularly	70.7	69.3	0.452

Statistically significant differences are in bold (*p* < 0.05).

**Table 2:** Differences in vegetable and fruit consumption, cooking skills, food skills, and food knowledge between the experimental and control groups.

	Control Group ^a^		Experimental Group ^a^		F ^b^	*p*-Value
	Mean (SD) Baseline (n = 82)	Mean (SD) Endpoint (n = 72)	Mean (SD) Baseline (n = 88)	Mean (SD) Endpoint (n = 77)		
Vegetable and fruit consumption (0 to 14 pts)	6.15 (4.37)	5.63 (4.62)	5.50 (4.12)	6.14 (4.50)	1.444	0.231
Boys	5.48 (4.18)	5.00 (4.57)	4.79 (3.87)	4.97 (4.28)	0.011	0.917
Girls	6.68 (4.47)	6.09 (4.50)	6.20 (4.32)	7.28 (4.52)	2.478	0.118
Cooking skills (0 to 91 pts)	27.95 (12.77)	33.14 (14.55)	36.52 (20.10)	53.32 (21.88)	**6.240**	**0.013**
Boys	27.59 (13.88)	32.05 (16.90)	27.65 (18.15)	44.32 (19.78)	**5.140**	**0.025**
Girls	28.05 (11.72)	34.12 (11.59)	43.40 (19.04)	59.13 (19.14)	1.255	0.265
Food knowledge (0 to 11 pts)	5.41 (1.61)	5.50 (1.85)	5.34 (1.98)	6.47 (1.54)	**4.861**	**0.028**
Boys	4.98 (1.64)	5.16 (1.79)	4.83 (2.00)	6.59 (1.40)	**5.368**	**0.022**
Girls	5.90 (1.46)	5.94 (1.85)	5.69 (1.91)	6.30 (1.68)	0.918	0.340
Food skills (0 to 53 pts)	12.79 (9.32)	16.46 (11.87)	15.57 (12.57)	21.57 (13.86)	0.789	0.375
Boys	10.37 (8.36)	16.21 (11.70)	10.23 (9.52)	16.41 (12.25)	0.210	0.648
Girls	15.00 (9.71)	16.30 (12.15)	20.00 (13.47)	25.53 (14.18)	0.857	0.356

^a^ Means in the columns are based on all data available at each measurement period. ^b^ Interaction term between time and group, with adjustments for school, age, gender, socioeconomic status, and ethnicity. Statistically significant results are in bold (*p* < 0.05).

**Table 3:** Differences in the odds of eating breakfast at least five times per week between the experimental and control groups.

	Control Group ^a^		Experimental Group ^a^		B ^b^	Exp(B) ^c^	95% CI	*p*-Value
	Percentage of Students Eating Breakfast Regularly Baseline (n = 82)	Percentage of Students Eating Breakfast Regularly Endpoint (n = 72)	Percentage of Students Eating Breakfast Regularly Baseline (n = 88)	Percentage of Students Eating Breakfast Regularly Endpoint (n = 77)				
Breakfast consumption	70.7	68.1	69.3	79.2	0.521	1.684	0.65, 4.36	0.282
Boys	65.9	55.3	70.0	82.4	0.926	2.525	0.67, 9.52	0.171
Girls	75.0	81.8	66.7	75.0	0.226	1.254	0.24, 6.65	0.791

^a^ Means in these columns are based on all data available at each measurement period. ^b^ Interaction term between time and group, with adjustments for school, age, gender, socioeconomic status, and ethnicity. ^c^ Odds ratio. Statistically significant results are in bold (*p* < 0.05).

## Data Availability

The dataset generated during this study is not publicly available to ensure confidentiality but is available from the corresponding author on reasonable request.
